# Pharmacokinetics and Metabolism of Ziritaxestat (GLPG1690) in Healthy Male Volunteers Following Intravenous and Oral Administration

**DOI:** 10.1002/cpdd.1021

**Published:** 2021-10-11

**Authors:** Eric Helmer, Ashley Willson, Christopher Brearley, Mark Westerhof, Stephane Delage, Iain Shaw, Ray Cooke, Sharan Sidhu

**Affiliations:** ^1^ Galapagos Biotech Limited Cambridge UK; ^2^ Quotient Sciences Nottingham UK; ^3^ Galapagos NV Mechelen Belgium; ^4^ Pharmaron Rushden UK

**Keywords:** ADME, IPF, mass balance, systemic scleroses, ziritaxestat

## Abstract

Ziritaxestat is a novel inhibitor of autotaxin, an enzyme responsible for the production of lysophosphatidic acid, the downstream signaling of which mediates responses to tissue injury and has been implicated in the pathogenesis of fibrotic conditions such as idiopathic pulmonary fibrosis and systemic sclerosis. This study (Clinical Trial Registration: NCT03787186) was designed to assess the absorption, distribution, metabolism, and excretion of orally administered 600‐mg ziritaxestat labeled with a carbon‐14 tracer (^14^C‐ziritaxestat). To understand the absolute bioavailability of ziritaxestat, an intravenous 100‐μg microdose, labeled with a microtracer amount of ^14^C radiation, was administered in a separate part of the study, following an unlabeled 600‐mg therapeutic oral dose of ziritaxestat. Six healthy male subjects completed each study part. The majority of the labeled oral dose was recovered in feces (77%), with a total mass balance of 84%. The absolute bioavailability of ziritaxestat was 54%. Ziritaxestat was the main (76%) circulating drug‐related product. There were 7 treatment‐emergent adverse events, all of which were considered mild and not considered to be related to the study drug.

Idiopathic pulmonary fibrosis (IPF) is a progressive fibrotic interstitial lung disease that can lead to loss of lung function and premature death.^1^, ^2^, ^3^ Current approved treatments for IPF are nintedanib, a tyrosine kinase inhibitor, and pirfenidone, an oral antifibrotic drug.^4^, ^5^ Systemic sclerosis is characterized by fibrosis of the skin and internal organs,^6^ with vasculopathy and inflammation preceding fibrosis.^7^ Current treatment of systemic sclerosis relies on immunomodulatory drugs such as methotrexate, as well as treatments aimed at easing symptoms or treating individual organ systems.^8^, ^9^ Autotaxin (ATX), also known as ectonucleotide pyrophosphatase‐phosphodiesterase 2, is a lysophospholipase D responsible for extracellular lysophosphatidic acid (LPA) production.^10^ Downstream signaling of LPA occurs via G protein–coupled receptors (LPA_1_‐LPA_6_), mediating responses to tissue injury, including excessive responses that lead to fibrosis.^11^ Specifically, links between LPA signaling through its receptor LPA_1_ have long been established in preclinical models of IPF and systemic sclerosis,^12^, ^13^, ^14^ with LPA_1_‐deficient mice protected from fibrosis in bleomycin models of lung and dermal fibrosis.^12^, ^14^ Increased ATX expression has been observed in the fibrotic skin of patients with systemic sclerosis, as well as in a bleomycin mouse model of systemic sclerosis.^15^ In the animal model, ATX inhibition reduced dermal fibrosis when initiated before or after fibrosis was established.^15^ Increased ATX and LPA levels have also been found in the lung tissues and bronchoalveolar lavage lung fluid, respectively, of patients with IPF.^12^, ^16^

Ziritaxestat is a novel ATX inhibitor^17^ that was in development as a treatment for IPF and systemic sclerosis.^18^ Phase 2 studies have been completed in patients with IPF^19^ and systemic sclerosis^20^; however, phase 3 trials of ziritaxestat in addition to standard‐of‐care treatment in patients with IPF were halted because the benefit‐risk profile of ziritaxestat was found to no longer support continuation of these trials. The pharmacokinetics (PK) of ziritaxestat have been reported separately.^18^ Ziritaxestat is rapidly absorbed and eliminated, with exposure to parent increasing in a dose‐proportional manner. In vitro human recombinant cytochrome P450 (CYP) studies indicated that ziritaxestat was primarily metabolized by CYP3A4 with minor contributions (maximum of 2.1%) to metabolism from other phase 1 enzymes. Increases in messenger RNA expression when 0.1 to 30 μM of ziritaxestat was incubated with human hepatocytes indicated that a 600‐mg once‐daily dose in humans may have the potential to induce CYP3A4 and CYP1A2. Weak inhibition of CYP2C8 and CYP3A4/5 was seen in in vitro studies with human liver microsomes and probe substrates with half maximal inhibitory concentrations of 2.1 and 3.7 μM, respectively. Strong, time‐dependent, irreversible inhibition potential was observed with CYP2C8‐mediated metabolism, with a half maximal inhibitory concentration fold shift of 2.49 following preincubation with ziritaxestat in the absence and presence of nicotinamide adenine dinucleotide phosphate. Five metabolites have been previously detected in human plasma and urine. These metabolites, resulting from hydrolysis, hydroxylation, addition of water, demethylation, or reduction, were all detected in plasma of either rat or dog, with both species used for general toxicity testing.

During the clinical development of ziritaxestat into phase 3, there was a need to further explore the absorption, distribution, metabolism, and excretion, and the absolute bioavailability properties, of orally administered ziritaxestat. The objectives of the present study were to assess the mass balance using carbon‐14 (^14^C)‐labeled ziritaxestat to better characterize the elimination pathways and biotransformation of ziritaxestat, to further characterize the PK of ziritaxestat and its main metabolites, to evaluate the absolute bioavailability of oral ziritaxestat, and to evaluate the safety and tolerability of ziritaxestat in healthy male volunteers. To achieve these objectives, a 2‐part study design was conceived, involving an intravenous (IV) microdose of ziritaxestat labeled with a microtracer amount of ^14^C radioactivity (part 1). Part 2 involved a therapeutic oral dose of ^14^C‐labeled ziritaxestat. Administration of radioactivity via both IV and oral routes allowed for estimation of key bioavailability parameters, namely, the fraction of drug absorbed (f_a_), the fraction of drug surviving gut metabolism (f_g_), and the fraction of drug surviving hepatic elimination (f_h_).

## Methods

### Study Design

This was a phase 1, 2‐part, sequential, open‐label study to evaluate the absolute bioavailability and absorption, distribution, metabolism, and excretion properties of ziritaxestat in healthy male subjects (Clinical Trial Registration: NCT03787186). Subjects were required to be nonsmokers, aged 30 to 64 years, with a body mass index (BMI) between 18 and 32 kg/m^2^ inclusive. Given the use of ionizing radiation in the study, subjects were excluded if they had been exposed to >5 mSv in the past 12 months or 10 mSv in the past 5 years. All medications (including over‐the‐counter and prescription medications and dietary, herbal, vitamin, and nutraceutical supplements) except occasional acetaminophen were not permitted for at least 2 weeks before the first dose of ziritaxestat and throughout the study. In both study parts, subjects fasted overnight for ≥10 hours. On the day of administration, the oral drug products were administered 30 minutes after the start of a standard breakfast. The study was conducted between November 2018 and January 2019 at Quotient Sciences (Nottingham, UK) in accordance with the clinical trial protocol, International Conference on Harmonisation of Technical Requirements for Registration of Pharmaceuticals for Human Use Good Clinical Practice guidelines, the Medicines for Human Use (Clinical Trials) regulations (2004) and amendments (2006, 2008), and the ethical principles outlined in the World Medical Association's Declaration of Helsinki and its amendments. The study was reviewed and approved by the London–Surrey Borders Research Ethics Committee (London, UK), the Administration of Radioactive Substances Advisory Committee, and the Medicines and Healthcare Products Regulatory Agency. All subjects provided written informed consent before any study‐related procedures were performed.

### Absolute Bioavailability

Part 1 of the study was designed to assess the absolute bioavailability of ziritaxestat. An IV microtracer, a well‐established technique, was used.^21^, ^22^ This design involved a 15‐minute infusion of a subtherapeutic 100‐μg IV ziritaxestat dose labeled with a microtracer (≤1000 nCi) of ^14^C radioactivity, dosed at an anticipated time to reach maximum plasma concentration (t_max_) of a preceding unlabeled therapeutic (600‐mg) oral dose (administered as three 200‐mg film‐coated tablets). The low‐dose IV formulation means that nonclinical local tolerability and IV toxicology studies can generally be avoided,^21^, ^22^ which can be preferable when developing a single, fit‐for‐purpose IV formulation. Administering the IV microdose at t_max_ of the unlabeled therapeutic (600‐mg) oral dose of ziritaxestat ensured that the PK of the microdose remained representative of the therapeutic dose.

Given the low (microgram) levels of ziritaxestat expected in plasma, liquid chromatography–accelerated mass spectrometry (LC‐AMS) was used to quantify levels of ^14^C‐ziritaxestat, whereas accelerated mass spectrometry (AMS) was used to quantify total radioactivity following the IV dose, achieving a lower limit of quantification (LLOQ) of 0.110 ng eq/mL and 0.0069 ng eq/mL, respectively. Concentrations of unlabeled parent in plasma were measured using liquid chromatography with tandem mass spectrometry (LC‐MS/MS), with an LLOQ of 1.00 ng/mL.

Subjects were admitted to the clinic on day –1 (the evening before dosing) and remained onsite until 72 hours after dosing. On the morning of day 1, 30 minutes after the start of a standard breakfast, subjects received a single oral dose of 600‐mg ziritaxestat, followed 3.25 hours later by a 15‐minute IV infusion of 100 μg of ^14^C‐ziritaxestat. Plasma samples for ziritaxestat concentrations were collected before dosing and at 0.25, 0.5, 1, 1.5, 2, 3, 3.25, 4, 5, 6, 8, 10, 12, 24, 48, and 72 hours after the oral dose. In addition, plasma samples for ^14^C‐ziritaxestat, total radioactivity, and metabolite profiling were collected at 3.25, 3.33, 3.5, 3.75, 4, 4.25, 4.75, 5.25, 6, 7, 8, 10, 12, 24, 48, and 72 hours after the oral dose.

### Absorption, Distribution, Metabolism, and Excretion

Part 2 of the study assessed the mass balance, routes of elimination, and metabolite profile of ziritaxestat following a single, oral 600‐mg dose (administered as two 300‐mg active pharmaceutical ingredients‐in‐capsule) of ^14^C‐ziritaxestat.

Subjects were admitted on the evening before dosing (day –1 of part 2), allowing a minimum 7‐day washout in oral drug administration between parts 1 and 2. On the morning of day 1, subjects received a single oral dose of ^14^C‐ziritaxestat, equivalent to 600 mg of ziritaxestat. Subjects could be discharged from day 8 (168 hours after dosing) as a group, provided every individual met 1 of the following 2 criteria: a mass balance recovery of >90% of the administered radiolabeled dose, or <1% of the administered radiolabeled dose collected in excreta (urine and feces) within 2 separate, consecutive 24‐hour periods. Subjects stayed in the clinical study center no longer than day 10 (216 hours after dosing).

Whole blood and plasma samples for total radioactivity and plasma sample concentrations of ziritaxestat were collected before dosing and at 0, 0.25, 0.5, 1, 1.5, 2, 3, 4, 6, 8, 10, 24, 48, 72, 96, 120, 144, 168, 192, and 216 hours after dosing. Plasma samples for metabolite identification and quantification were collected before dosing and at 1, 3, 6, 24, 48, 72, 96, and 144 hours after dosing. Urine and fecal samples for total radioactivity, metabolite identification, and quantification were collected before dosing through to 216 hours after dosing. Structural identification was attempted on any radioactive component that represented ≥10% of the circulating radioactivity in plasma or that accounted for >10% of the dose in ≥1 fecal and urine samples.

Total radioactivity was measured via liquid scintillation counting, achieving an LLOQ of 306 ng eq/mL (plasma), 216 ng eq/mL (whole blood), 17 ng eq/g (urine), and 161 ng eq/g (feces). Concentrations of parent in plasma were measured using LC‐MS/MS, with an LLOQ of 1.00 ng/mL.

### Bioanalysis

Concentrations of ziritaxestat in human lithium heparin plasma samples were determined for both study parts by using an LC‐MS/MS method validated according to the requirements of the US Food and Drug Administration's Guidance for Industry and the European Medicines Agency's guidance on bioanalytical methods. The method involved isolating ziritaxestat and ziritaxestat‐d8 (internal standard) from 20 μL of human lithium heparin plasma by solid‐phase extraction performed on a CUBCX1 100‐mg 1‐mL cartridge (Screening Devices, Amersfoort, Netherlands). The extract sample was then evaporated to dryness under a stream of nitrogen at +50°C. The residue was reconstituted with 0.3 mL of a mixture of 25% acetonitrile in purified water containing 0.01% of ammonium hydroxide. Ten microliters of the clear supernatant were injected into the high‐performance liquid chromatography system (Shimadzu, Kyoto, Japan), operating in isocratic elution mode. Chromatographic separation was performed on an XBridge C18 column (100 × 4.6 mm, 3.5 μm; Waters Corp., Milford, Massachusetts). The aqueous mobile phase consisted of a mixture of 46.75% acetonitrile in purified water containing 0.02% of ammonium hydroxide. An API 4000 LC‐MS/MS system (SCIEX, Toronto, Canada) equipped with a TurboIonSpray probe operating in multiple‐reaction monitoring mode in positive mode was used for quantification. The precursor‐to‐product ion pairs at the mass‐to‐charge ratio were 589 to 256 and 597 to 264 for ziritaxestat and its internal standard, respectively.

Calibration curves in plasma were linear over the range of 1 to 1000 ng/mL, with 1/×2 as weighting factor.

In each analytical run, duplicate quality control (QC) samples at low (3.00 ng/mL), medium (400 ng/mL), and high (800 ng/mL) concentrations were analyzed along with the study samples. Overall (mean) precision (expressed as coefficient of variation [CV]) and accuracy (expressed as percent bias) were calculated at each concentration level and were ≤6.8% CV and within –7.9% to –1.7% bias. Incurred sample reanalysis (ISR) was successfully performed during the study, demonstrating the reliability of the obtained results.

The mean precision and accuracy for the low, medium, and high QC samples were ≤5.3% CV and within –2.3% to 1.7% bias, respectively, indicating that the method performed reliably during the analysis of stability samples.

Plasma samples were analyzed within the time frame where the stability of study samples was covered by the stability data available.

### Accelerated Mass Spectrometry

Total radioactivity plasma concentrations for part 1 were determined using AMS analysis on a 1‐MV multielement accelerated mass spectrometer, model 4110 Bo (High Voltage Engineering Europa, Amersfoort, the Netherlands). A total of 5 μL of plasma (diluted or undiluted) was transferred to tin foil cups. Samples were dried under a stream of nitrogen and placed in the elemental analyzer that acted as an autosampler and combustion device for the AMS. Plasma results were corrected for individual background values and were next converted to nanogram equivalents per milliliter using the specific activity of the dose formulation.

In each AMS batch (including LC‐AMS), QC samples were included with a minimum of 3 replicates. These samples were prepared by spiking blank pooled plasma with a known amount of ^14^C (derived from ^14^C‐acetaminophen). The ^14^C/^12^C ratios were determined by 5‐fold analysis using AMS before the start of the study sample analysis.

The QC results fulfilled the requirements for all analytical runs. The average ^14^C/^12^C ratio of the QC samples in a batch did not deviate >15% from the nominal value. In addition, the CV was <15% in all runs.

### Liquid Chromatography–Accelerated Mass Spectrometry

^14^C‐ziritaxestat plasma concentrations for part 1 were determined by using LC‐AMS. The LC‐AMS method was previously qualified according to the European Bioanalysis Forum recommendation in the range of 10.4 to 1037 mBq/mL,^23^ in which the linearity, precision and accuracy, selectivity, carryover, and dilution integrity were successfully assessed, demonstrating the reliability of the entire analytical procedure.

Plasma samples were extracted before injection into the ultra‐performance liquid chromatography system by using 100 μL of plasma sample mixed with 600 μL of precipitation solvent.

During the ultra‐performance liquid chromatography analysis, the ziritaxestat (and ^14^C‐ziritaxestat) elution fraction was collected for each injection in 96‐well plates. The fractions were dried under a stream of nitrogen. Residues were reconstituted in 50 μL of acetonitrile, transferred to a tin foil cup, and dried under nitrogen. Samples were placed in the elemental analyzer that acted as an autosampler and combustion device for the AMS (the same equipment as used for the total radioactivity analyses).

^14^C/^12^C isotope ratios of the fractions collected were converted to megabecquerels per milliliter by plotting these values on the linear calibration line established in each analytical run with calibrator samples prepared at 8 levels. A weighting factor of 1/×2 was applied on the regression model. The parent drug–related ^14^C concentrations in the plasma samples (megabecquerels per milliliter) were also converted to nanogram equivalents per milliliter, based on the specific activity of the compound.

The ISR fulfilled the acceptance criteria on the basis that 11 of 12 (92%) of the ISR samples had differences <20% when comparing the original and ISR result with the averaged value.

### Pharmacokinetic Analysis

PK analysis was performed using standard noncompartmental methods (Phoenix WinNonlin version 9.4; Pharsight, Certara, Princeton, New Jersey). PK parameters included maximum plasma concentration (C_max_), t_max_, area under the plasma concentration–time curve (AUC) to the last sampling time point with quantifiable levels of parent (AUC_t_), AUC to 24 hours (AUC_24_), AUC extrapolated to infinity, total body clearance of parent following IV administration (CL), terminal elimination half‐life (t_1/2_), volume of distribution of parent following the IV route (V_d(area)_), and absolute bioavailability (F). In part 2, the following mass balance parameters were determined: cumulative amount of total radioactivity excreted in urine up to t, cumulative amount of total radioactivity excreted in feces up to t, amount of total radioactivity excreted in urine as a percentage of administered radioactivity, and amount of total radioactivity excreted in feces as a percentage of administered radioactivity. AUC was calculated by the linear up‐logarithmic down‐trapezoid rule, CL was calculated as dose/AUC, Vd_(area)_ was calculated as CL/λ_z_, and F was calculated as dose‐normalized ziritaxestat oral AUC/^14^C‐ziritaxestat IV AUC.

The fraction of ziritaxestat absorbed was calculated from 100% minus the percentage of parent drug in feces and expressed as a percentage of the total dose. f_h_ was calculated based on Fh=1−Eh (where Eh [hepatic extraction ratio] was estimated as clearance over average hepatic flow rate [Qh], or $\frac{Cl_{IV}}{Qh}$), using a nominal value of 1450 mL/min for average hepatic blood flow.^24^, ^25^ Contribution to clearance from nonhepatic routes was assumed to be minimal. f_g_ was estimated assuming Fg=F/Fa/Fh.

### Safety Assessments

Safety assessments included adverse events (AEs), clinical laboratory tests (clinical chemistry, hematology, urinalysis), vital signs, 12‐lead electrocardiograms (ECGs), and physical examinations. AEs were recorded from the first dose until study discharge, and at the follow‐up call. Any adverse experiences recorded between the time of informed consent and the first dose were recorded with the medical history. AEs and medications were coded using the Medical Dictionary for Regulatory Activities version 21.1.

### Statistical Analysis

This was an exploratory study. The numbers of subjects per cohort are consistent with common practice for this study type and were considered suitable to achieve the study objectives. Descriptive statistics for safety data are presented as cases (%). PK parameters, C_max_, AUC, t_1/2_, CL or CL/F, Vd_(area)_, and F are presented as arithmetic means with standard deviation (SD). T_max_ is expressed as the median (range). Subject disposition (age and BMI) is presented as median (range). All statistical analyses were performed using SAS version 9.4 or higher (SAS Institute, Cary, North Carolina), and/or Phoenix WinNonlin version 8 software.

## Results

### Pharmacokinetics

**Figure 1:**
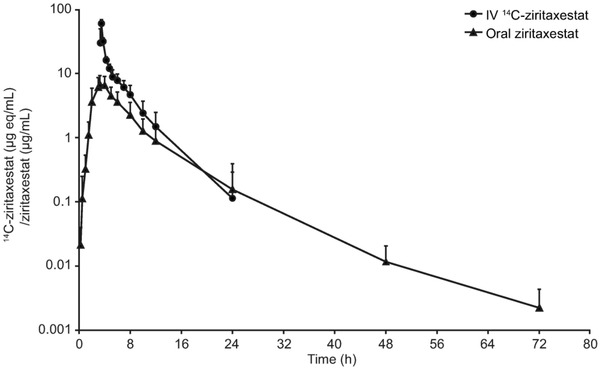
Dose‐normalized ^14^C‐ziritaxestat and ziritaxestat plasma concentration: mean (SD) concentrations over time (log‐linear scale). ^14^C‐ziritaxestat concentrations have been dose normalized to a 600‐mg dose. ^14^C, carbon‐14; eq, equivalent; IV, intravenous; SD, standard deviation.

In part 1, following a 100‐μg IV infusion of ^14^C‐ziritaxestat, a mean (SD) C_max_ of 10.2 ng eq/mL (1.4) was achieved, with a mean (SD) AUC_t_ of 12.4 ng eq • h/mL (3.8) (Table 1). The mean (SD) t_1/2_ recorded was 2.8 hours (1.4). A mean CL of 8.2 L/h was also recorded with a mean volume of distribution of 29.9 L. The absolute bioavailability of the 600‐mg oral dose of ziritaxestat was estimated to be 54%, calculated from oral and IV AUC_t_. IV ^14^C‐ziritaxestat accounted for about 60% of circulating plasma total radioactivity. The elimination t_1/2_ for plasma total radioactivity was longer than ^14^C‐ziritaxestat at 47.8 hours (Table 1, Figure 1).

**Table 1:** Plasma PK Parameters of Oral Ziritaxestat, IV ^14^C‐Ziritaxestat, and Total Radioactivity Following Oral Administration of 600 mg Ziritaxestat and a 15‐Minute IV Infusion of 100 μg ^14^C‐Ziritaxestat (PK Analysis Set)

PK Parameter,^a^ Unit	Ziritaxestat Oral 600 mg (n = 6)	^14^C‐Ziritaxestat IV Infusion 100 μg ^14^C‐Ziritaxestat (n = 6)	Total Radioactivity IV Infusion 100 μg ^14^C‐Ziritaxestat (n = 6)
t_max_, h	3.25 (2.00‐4.00)	0.25 (0.23‐0.25)	0.25 (0.23‐0.25)
C_max_, μg/mL or ng eq/mL^b^, ^c^	8.4 (1.7)	10.2 (1.4)	7.8 (0.9)
AUC_t_, μg • h/mL or ng eq • h/mL^b^	40.0 (13.4)	12.4 (3.9)	20.5 (4.7)
AUC_∞_, μg • h/mL or ng eq • h/mL^b^	40.0 (13.4)	13.2 (4.1)	NC^d^
t_1/2_, h	7.0 (1.6)	2.8 (1.4)	47.8 (25.6)^d^
CL or CL/F, L/h	16.3 (4.6)	8.2 (2.2)	…
V_d(area)_, L	…	29.9 (7.5)	…
F AUC_∞_, %	51.1 (4.8)	…	…
F AUC_t_, %	54.4 (5.2)	…	…

^14^ C, carbon‐14; AUC_∞_, area under the plasma concentration–time curve from time 0 to infinity; AUC_t_, area under the plasma concentration–time curve from time 0 to time t; CL, total body clearance; C_max_, maximum plasma concentration; eq, equivalent; F, absolute bioavailability; IV, intravenous; NC, not calculated; PK, pharmacokinetic; SD, standard deviation; t_1/2_, terminal elimination half‐life; t_max_, time to reach maximum plasma concentration; V_d(area)_, volume of distribution following IV route.

^a^ Results are expressed as arithmetic mean (SD) except for t_max_, which is expressed as median (range).

^b^ Microgram units for ziritaxestat parameters after oral dosing, and nanogram units for ^14^C‐ziritaxestat and total radioactivity parameters.

^c^ For IV infusion, C_max_ corresponds to the concentration at the end of the infusion.

^d^ Terminal slopes for total radioactivity plasma concentration versus time profiles were reliably determined in 5 of 6 subjects; in the remaining subject, the terminal slope could not be reliably determined.

**Figure 2:**
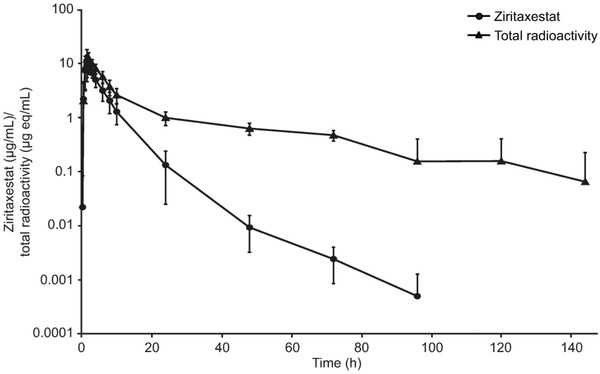
Ziritaxestat and total radioactivity plasma concentration: mean (standard deviation) plasma concentration over time (log‐linear scale). eq, equivalent.

In part 2, following a 600‐mg oral dose of ^14^C‐ziritaxestat, the mean C_max_ (SD) was 10.7 μg/mL (4.4), with a mean (SD) exposure (AUC_t_) of 49.1 μg • h/mL (17.6). The mean (SD) t_1/2_ recorded was 10.6 hours (3.6). Exposure to ^14^C‐ziritaxestat accounted for about 56% of circulating plasma total radioactivity. This was calculated using AUC_24_, as total radioactivity counts from 48 hours onwards were approaching background levels of radioactivity (Table 2, Figure 2).

**Table 2:** Plasma and Whole Blood PK Parameters Following a Single 600‐mg Dose of Oral ^14^C‐Ziritaxestat (PK Analysis Set)

PK Parameter,^a^ Unit	Ziritaxestat Plasma (n = 6)	Total Radioactivity Plasma (n = 6)	Total Radioactivity Whole Blood (n = 6)
t_max,_ h	1.75 (1.00‐2.00)	2.00 (1.50‐3.00)	2.00 (1.00‐3.00)
C_max_, μg/mL^b^	10.7 (4.4)	13.9 (5.1)	8.65 (3.1)
AUC_t,_ μg • h/mL^b^	49.1 (17.6)	127.4 (46.9)	162.24 (24.0)
AUC_24_, μg • h/mL^b^	47.9 (16.8)	85.7 (24.6)	58.4 (15.0)
AUC_∞_, μg • h/mL^b^	50.5 (19.3) [n = 5]^c^	NC^d^	NC^e^
t_1/2_, h	10.6 (3.6) [n = 5]^c^	54.4 (14.5) [n = 4]^d^	NC^e^
CL/F, L/h	13.2 (4.6) [n = 5]^c^	NC	…
CL_R_, L/h	…	0.3 (0.1)	…

^14^ C, carbon‐14; AUC_∞_, area under the plasma concentration–time curve from time 0 to infinity; AUC_24_, area under the plasma concentration–time curve to 24 hours; AUC_t_, area under the plasma concentration–time curve from time 0 to time t; CL, total body clearance; CL_R_, renal clearance; C_max_, maximum plasma concentration; F, absolute bioavailability; NC, not calculated; PK, pharmacokinetic; SD, standard deviation; t_1/2_, terminal elimination half‐life; t_max_, time to reach maximum plasma concentration.

^a^ Results are expressed as arithmetic mean (SD) except for t_max_, which is expressed as median (range).

^b^ Microgram equivalent units for total radioactivity parameters.

^c^ Terminal slopes for ziritaxestat plasma concentration versus time profile could not be reliably estimated in 1 subject.

^d^ Terminal slopes for total radioactivity plasma concentration versus time profile could not be reliably estimated in 2 subjects.

^e^ Terminal slopes for total radioactivity whole concentration versus time profile could not be reliably estimated in 5 subjects.

### Mass Balance

Following a single oral dose of 600 mg ^14^C‐ziritaxestat, an average of 84.1% of the administered radioactivity was recovered in excreta by the end of the sampling period (288 hours after dosing). The majority (77.4%) of the radioactivity was recovered in feces, with 6.7% eliminated in urine. Within the first 48 hours, 26% of total radioactivity was recovered in feces (Table 3).

**Table 3:** Mean Amount of Total Radioactivity Excreted in Urine and Feces Following a Single Oral Dose of ^14^C‐Ziritaxestat (Mass Balance Analysis Set)

Collection Interval (h)	Urine A_et_(% of Administered Radioactivity) (n = 6)	Feces A_ft_(% of Administered Radioactivity) (n = 6)	Total A_t_(% of Administered Radioactivity) (n = 6)
0‐4^a^	0.45	…	0.45
4‐8^a^	1.26	…	1.26
8‐12^a^	1.65	…	1.65
12‐24^a^/0‐24^b^	1.27	5.40	6.67
24‐48	0.93	20.38	21.31
48‐72	0.43	14.83	15.26
72‐96	0.24	23.92	24.16
96‐120	0.16	7.76	7.92
120‐144	0.09	2.52	2.61
144‐168	0.07	1.00	1.07
168‐192	0.07	0.66	0.73
192‐216	0.04	0.47	0.51
216‐240	NC	0.34	0.37
240‐264	NC	0.12	0.12
264‐288	NC	NC	NC
	Ae% (SD)	Af% (SD)	At% (SD)
	6.68 (0.77)	77.42 (18.44)	84.1 (18.40)

^14^ C, carbon‐14; Ae%, amount of total radioactivity excreted in urine as a percentage of administered radioactivity; A_et_, amount of total radioactivity excreted in urine up to time t; Af%, amount of total radioactivity excreted in feces as a percentage of administered radioactivity; A_ft_, amount of total radioactivity excreted in feces up to time t; A_t_, amount of total radioactivity excreted in urine and feces; At%, amount of total radioactivity excreted in urine and feces as a percentage of administered radioactivity; NC, not calculated; SD, standard deviation.

^a^ Collection interval in urine.

^b^ Collection interval in feces.

### Metabolite Identification

No single component other than ^14^C‐ziritaxestat accounted for >10% of circulating radioactivity in plasma. According to the mass spectrometry analysis, parent represented 76% of the circulating radioactivity in plasma, 28% of drug‐related material in urine, and 18% in feces. The major components identified are detailed in Table 4.

**Table 4:** Summary of Major Metabolites Observed in Feces and Plasma

Component	Retention Time (Minutes)	Mean Feces Region of Interest (%)	Mean Plasma Region of Interest
M620: Ring‐opened acid metabolite	22.5	25.3	NA
M590: Glycine conjugate of N‐dealkylated ziritaxestat	23.0	25.3	NA
M533: N‐dealkylated ziritaxestat	23.2	25.3	NA
M620: Ring‐opened azetidin‐3‐ol,mono‐hydroxy, desaturated (or keto) metabolite of ziritaxestat	27.1	19.4	NA
M604: Mono‐hydroxy ziritaxestat	37.2	17.9	7.9
M592: Ring‐opened azetidin‐3‐ol metabolite of N‐demethylated ziritaxestat	37.6	17.9	3.3
Ziritaxestat	75.5	17.6	76.2

NA, not applicable (identification not applicable in this sample).

### Oral Bioavailability

**Figure 3:**
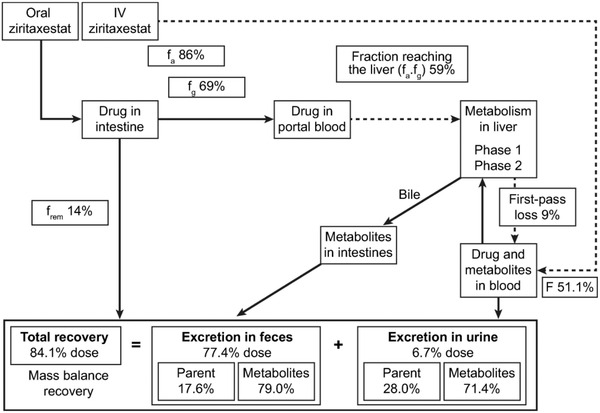
Fate of ziritaxestat following oral administration. F, bioavailability; f_a_, fraction of drug absorbed; f_g_, fraction of drug surviving gut metabolism; f_rem_, fraction of drug remaining to be eliminated; IV, intravenous.

It was assumed that the drug does not undergo biliary recirculation (this was supported by the absence of secondary parent peaks following oral administration) and that there would be minimal instability of unabsorbed parent in the gastrointestinal tract. A mean of 17.6% of the total radioactivity recovered in feces was parent, which equated to approximately 14% of the total dose and a f_a_ of 86%; f_h_ was estimated to be 91%. As both f_a_ and f_h_ had been calculated, f_g_ was estimated to be 69%. Based on the estimated f_a_ and f_g_ values of 86% and 65%, respectively, the estimated fraction of drug reaching the hepatic portal vein f_a_.f_g_ was 59% (Figure 3).

### Subjects

A total of 6 subjects were enrolled and completed part 1 of the study. Two subjects did not participate in part 2, due to personal reasons between part 1 and part 2, and therefore an additional 2 subjects were enrolled to ensure that 6 subjects completed both study parts. In part 1, the median age was 59.5 years (Table 5) and the median BMI was 27.55 kg/m^2^. In part 2, the median age was 56.0 years, and the median BMI was 27.35 kg/m^2^. All subjects enrolled in the study were White men.

**Table 5:** Demographics and Baseline Characteristics of Subjects in Part 1 and Part 2 (Safety Analysis Set)

Characteristic, Unit	Part 1 (n = 6)	Part 2 (n = 6)
Age, y	54.2 (11.2)	54.3 (8.7)
Height, cm	174.0 (5.1)	174.5 (8.3)
Weight, kg	84.3 (9.6)	84.8 (14.1)
BMI, kg/m^2^	27.8 (2.4)	27.7 (2.5)
Race (White), n (%)	6 (100)	6 (100)

BMI, body mass index.

Data shown as arithmetic mean (standard deviation) unless indicated otherwise.

### Safety

There were no serious AEs or AEs leading to discontinuation or interruption of dosing during the study. A total of 7 treatment‐emergent AEs (TEAEs) were reported in 5 subjects: 3 subjects had TEAEs of upper respiratory tract infection, pain in extremity, and headache in part 1, while 3 subjects reported TEAEs of nasopharyngitis, arthralgia, headache, and pruritus in part 2. The subject who reported headache in part 1 also reported headache in part 2. All TEAEs were mild in intensity and not treatment related as assessed by the investigator. There were no time‐dependent trends in (mean/median) actual values or changes from baseline observed based on laboratory, vital signs, or ECG parameters. There were no clinically meaningful effects on laboratory, vital signs, or ECG parameters identified.

## Discussion

This phase 1 healthy‐volunteer study was designed to generate extensive PK data to provide required regulatory data, as well as a detailed pathway for the fate of ziritaxestat following oral administration. Ziritaxestat is an ATX inhibitor that was in development for the treatment of IPF and systemic sclerosis.^19^ Both indications have a limited choice of treatments available, with options mostly focused on symptomatic treatment rather than inhibiting or reversing the pathway that causes fibrosis.^5^, ^26^ Ziritaxestat represented a novel inhibitor targeting a promising pathway for the inhibition of ATX and reversal of fibrosis; however, development was halted during large‐scale efficacy trials following a review of the benefit‐risk profile.^19^, ^27^

The absolute bioavailability of oral ziritaxestat was estimated to be 51%, with low hepatic extraction ratio (9%) demonstrating that ziritaxestat is a low hepatic extraction drug.^28^ The fraction of drug surviving absorption was estimated on the basis of the amount of parent recovered in feces, on the assumption that minimal parent was eliminated into the duodenum via the bile duct. If parent had been eliminated into the bile duct, it would be expected that secondary peaks would have been visible on the plasma concentration‐by‐time graphs, as parent is steadily reabsorbed, a typical marker for enterohepatic reabsorption.^29^ In the absence of this marker, the f_a_ was estimated to be 86%, meaning that only 14% of the drug was eliminated via unabsorbed oral dose. This also reflects the limited amount of radiation recovered in feces between 0 and 48 hours, the expected interval for complete gastric transit.^30^ With f_a_ and f_h_ estimated, the f_g_ could be estimated at 65%, the lowest fraction of the 3 key bioavailability parameters. These estimations are indicative of a low hepatic extraction drug that is primarily extracted by gut metabolism. This is reflected by nonclinical in vitro data suggesting that ziritaxestat is primarily metabolized by CYP3A4, the most prevalent CYP enzyme within the gut.^31^

IV ^14^C‐ziritaxestat represented 60% of circulating plasma total radioactivity, indicating that the parent is the main circulating species in plasma. To calculate the percentage of parent of total radioactivity following oral ^14^C‐ziritaxestat, AUC_24_ was used, as total radioactivity counts 48 hours inclusive and beyond were approaching levels of background radioactivity. This resulted in parent accounting for 56% of the circulating total plasma radioactivity in part 2, as derived from liquid scintillation counting and LC‐MS/MS data. The components identified in plasma indicated that parent was again the main circulating drug‐related product, representing about 76% of the elucidated material in plasma, as measured from the metabolite profile data.

A shorter mean t_1/2_ for ^14^C‐ziritaxestat was seen following IV administration in comparison with oral administration. While a shorter t_1/2_ following an IV dose compared with an extravascular dose can sometimes be indicative of absorption limiting the elimination rate, or flip‐flop kinetics,^32^ it is possible that despite the much higher sensitivity of the AMS method, the shorter IV t_1/2_ was due to an inability to quantify ^14^C‐ziritaxestat for the same duration of time as the oral dose.^33^ Caution, therefore, should be used when interpreting the shorter IV parent t_1/2_ and subsequent extrapolated AUC. As the IV microdose was administered at t_max_ of an unlabeled 600‐mg oral dose, it is unlikely that the difference in t_1/2_ is due to a concentration effect. The longer total radioactivity t_1/2_ compared with parent t_1/2_ seen following both IV and oral administration of ^14^C‐ziritaxestat indicates the formation and presence of metabolites with longer t_1/2_ than parent. The presence of metabolites with longer elimination t_1/2_ may also explain the sustained presence of total radioactivity seen in both the oral and IV administration. The presence of metabolites with long t_1/2_ should be accounted for when considering multiple‐dose studies in case of accumulation of active moieties, although no metabolite that was ≥10% of circulating radioactivity was discovered in plasma. Exposure and C_max_ following oral doses in parts 1 and 2 were consistent with previously reported PK data from the first‐in‐human study for ziritaxestat.^18^ While the elimination t_1/2_ reported in the current study was longer in both study parts compared with the first‐in‐human study, the variability reported was also higher, possibly related to a smaller sample and a wider range of demographics for subjects enrolled in this study.

Following administration of oral ^14^C‐ziritaxestat, 77.4% of total radioactivity was recovered by fecal excretion, with 26% of total radioactivity recovered in feces in the first 48 hours after dosing. This is indicative that fecal excretion of the drug product was predominantly due to biliary elimination, rather than direct elimination of unabsorbed drug product.^30^ All TEAEs were mild in intensity and were not considered to be treatment related.

As is standard practice when dosing ionizing radiation to healthy volunteers for drugs that are not indicated for female‐only therapies, only male subjects could participate. While there has been no indication that ziritaxestat demonstrates a sex effect, the results of this study should be reviewed in the context of a homogeneous population. To minimize the number of subjects exposed to ionizing radiation, only 6 subjects were dosed.

The data produced from this study demonstrated that ziritaxestat is the main circulating drug‐related product with an absolute bioavailability of 54. The oral ziritaxestat PK observed were consistent with prior experience with this molecule.^18^ Dosing an IV form of ziritaxestat allowed for estimation of key PK parameters, such as CL and Vd_(area)_, which up to this point have only been estimated as a function of absolute bioavailability following administration of oral doses. The data from this study, including the PK, mass balance, and metabolism of ziritaxestat, may be useful for future drug development programs.

## Conclusions

Following oral administration of 600 mg of radiolabeled ziritaxestat, unchanged ziritaxestat in plasma accounted for 76% of the total radioactivity. This is consistent with low hepatic extraction (9%) and the absence of metabolite accounting for ≥10% of the circulating radioactivity. Ziritaxestat was well absorbed (at least 86%) and showed good oral absolute bioavailability (54). Approximately 77.4% of the total radioactivity was recovered in feces and about 6.7% in urine. Given the almost complete absorption, this high percentage recovered in feces indicates that ziritaxestat is eliminated mainly via the biliary route.

## Conflicts of Interest

Eric Helmer is an employee of Galapagos Biotech. Ashley Willson, Iain Shaw, and Sharan Sidhu are employees of Quotient Sciences. Mark Westerhof and Stephane Delage are employees of Galapagos NV. Christopher Brearley was an employee of Galapagos NV at the time the work was conducted. The study was conducted at Quotient Sciences. A clinical research agreement was in place, with standard stipulations regarding the ownership and publication of the data. Ray Cooke is an employee of Pharmaron. Pharmaron were responsible for the determination of mass balance (measurement of total radioactivity in excreta, blood, and plasma) and the metabolite analysis for part 2.

## Funding

This study was sponsored by Galapagos NV (Mechelen, Belgium).

## Author Contributions

Eric Helmer was involved in study conceptualization; Eric Helmer, Christopher Brearley, Mark Westerhof, and Stephane Delage were involved in study design and methodology; Christopher Brearley, Mark Westerhof, and Sharan Sidhu were involved in the investigation; Sharan Sidhu was involved in data curation; Eric Helmer, Ashley Willson, Stephane Delage, Iain Shaw, and Ray Cooke were involved in formal data analysis; Eric Helmer, Ashley Willson, Christopher Brearley, Stephane Delage, Iain Shaw, and Ray Cooke were involved in data visualization; and Eric Helmer, Ashley Willson, Christopher Brearley, Stephane Delage, Iain Shaw, Ray Cooke, and Sharan Sidhu were involved in data interpretation. The first full draft of the manuscript was written by Ashley Willson, and all authors commented on previous versions of the manuscript. All authors read and approved the final manuscript.
